# Comparison of three plate system for lateral malleolar fixation

**DOI:** 10.1186/1471-2474-15-360

**Published:** 2014-10-30

**Authors:** ZeYu Huang, Lei Liu, ChongQi Tu, Hui Zhang, Yue Fang, TianFu Yang, FuXing Pei

**Affiliations:** Department of Orthopaedics, West China Hospital, Sichuan University, 37# Guoxue Road, Chengdu, 610041 People’s Republic of China

**Keywords:** Lateral malleolar fracture, One-third tubular plate, Locking compression plates, Minimally invasive plate osteosynthesis (MIPO) technique, Complications

## Abstract

**Background:**

This study was to compare clinical and radiographic outcomes with three different implants and evaluate the effectiveness of minimally invasive plate osteosynthesis (MIPO) technique for the distal fibular fractures.

**Methods:**

We performed a retrospective cohort single-surgical team single-facility study between 2000 and 2011. 147 patients receiving surgical interventions for closed, displaced distal fibular fractures were included. Based on the different implants, patients were divided into three groups: Group A: one-third tubular plate; Group B: locking compression (LCP) metaphyseal plate; Group C: LCP distal fibula plate. Clinical and radiographic outcomes were compared among the three groups.

**Results:**

Totally, we found that patients in Group C had significant higher functional scores than those in Group A (p1 = 0.004; p2 = 0.002) (p1 stands for the p value for Olerud & Molandar Score, p2 stands for the p value for American Orthopaedic Foot & Ankle Society score). The healing time was significant less in Group C than that in Group A (p < 0.0001) and Group B (p < 0.0001). Subgroup analysis showed that: (1) For Weber A fracture, the functional scores of the Group C were higher than those in Group A (p1 = 0.020; p2 = 0.029) and B (p1 = 0.020; p2 = 0.034). (2) For Weber B fracture, the functional scores of the Group B (p1 = 0.033; p2 = 0.030) and C (p1 = 0.027; p2 = 0.017) were higher than those in Group A. No significant differences were observed in terms of the ankle range of motion, reduction accuracy and complication rate.

**Conclusions:**

Our study demonstrated using LCP metaphyseal plate in patients associated with lateral malleolar fracture could achieve significantly better OMS & AOFAS scores and less healing time than using one-third tubular plate. Specifically, For Weber A fracture, LCP distal fibula plate is much better than one-third tubular plate and LCP metaphyseal plate. While for Weber B fracture, LCP distal fibula plate and LCP metaphyseal plate are better than one-third tubular plate. As to the complications, using MIPO technique in patients with distal fibular fractures is at least comparable to the traditional one.

**Electronic supplementary material:**

The online version of this article (doi:10.1186/1471-2474-15-360) contains supplementary material, which is available to authorized users.

## Background

Ankle fractures are considered the most common injuries in clinical practice [1, 2], of which the distal fibular fractures have the highest incidence [3]. Even though no consensus has been reached on surgical intervention of the distal fibular fractures, many centers take it as a routine practice [4, 5]. Theoretically, on the one hand the patients can gain better control of limb rotation and anatomical alignment [6] by fixing the fibula, on the other hand, because of the anatomical features of the distal fibula, surgical interventions are always associated with complications such as nonunion, malunion, posttraumatic osteoarthritis and infection [7].

**Figure 1:**
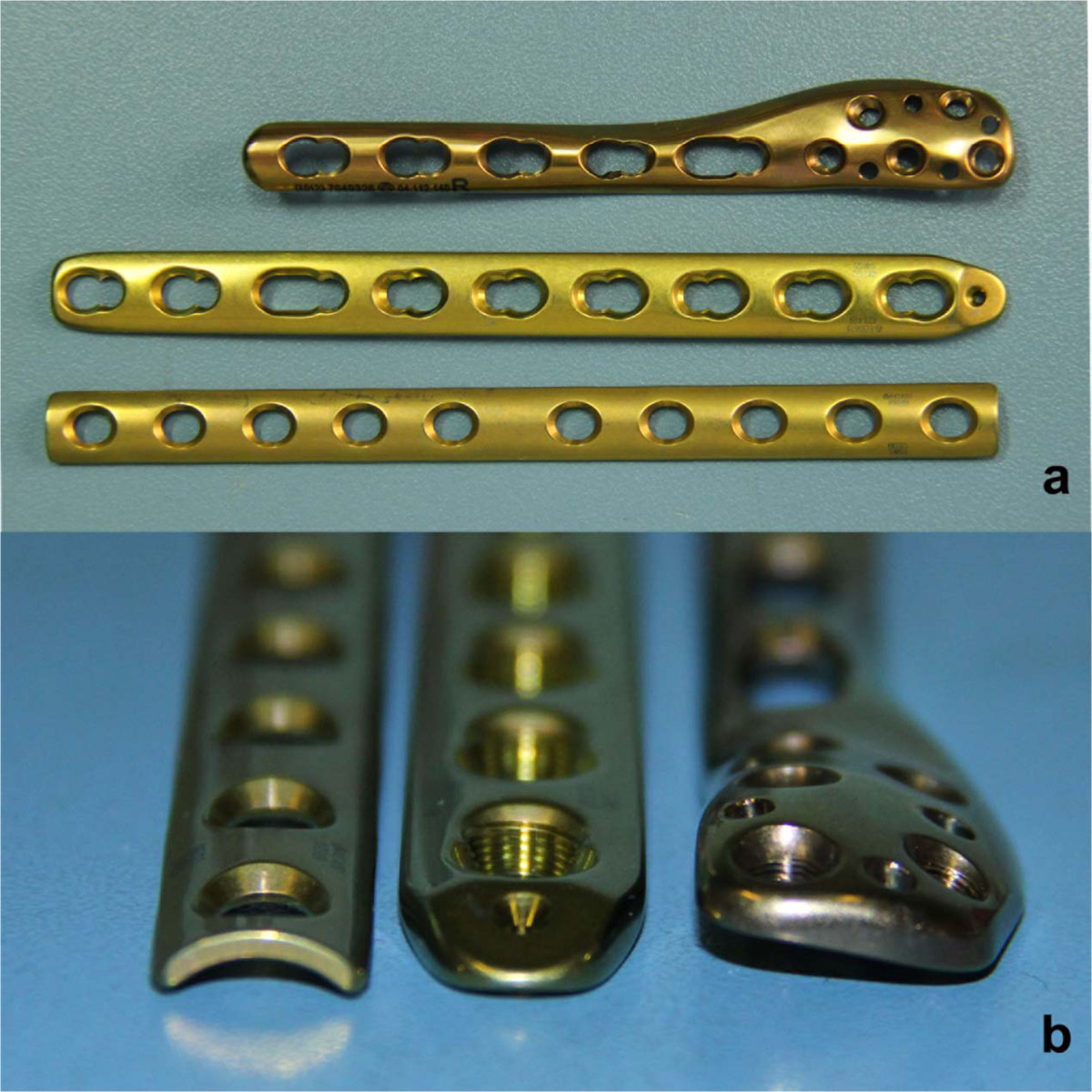
**Profile of the different plate types (a and b from left to right: conventional one-third tubular plate; a LCP metaphyseal plate; a LCP distal fibula plate).**

Nowadays, due to the stable fixations and capability of using minimally invasive plate osteosynthesis (MIPO) technique, the locking compression plates (LCPs) have been used for the treatment of various fractures [8, 9], as well as the distal fibular fractures. There are two different widely-used LCPs: one is LCP metaphyseal plate, the other is LCP distal fibula plate. To our knowledge, few literatures have compared these two different implants or compared with the one-third tubular (non-locking plates) for the treatment of distal fibular fractures (Figure 1a and b).

In order to compare the clinical effectiveness of the three different plates and find out the optimal indications for each implants, we designed this retrospective cohort study. We compared both the clinical and radiographic outcomes in the patients with closed, displaced distal fibular fracture managed with the three different implants matched by age, BMI and the classifications of the fractures.

## Methods

### Study design

**Figure 2:**
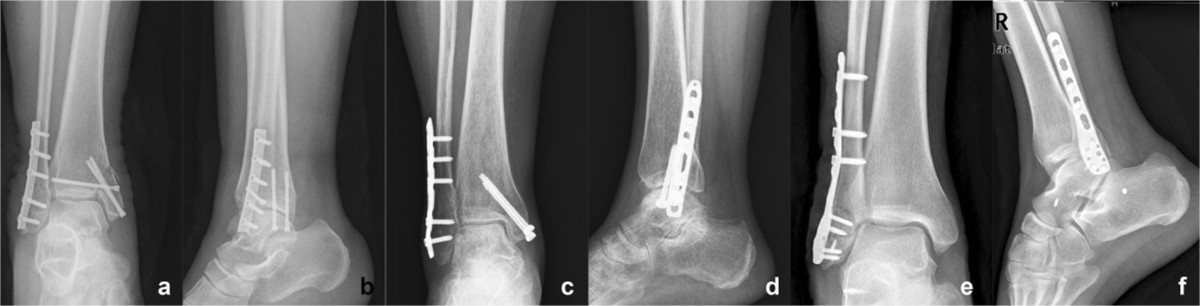
**Different plate types used in the current study. (a&b)** Conventional one-third tubular plate, **(c&d)** LCP metaphyseal plate, **(e&f)** LCP distal fibula plate.

This retrospective study was based on data collected in our prospective database and approved by the Institutional Review Board of West China Hospital of Sichuan University. From 2000 to 2011, 214 patients were treated by internal fixation for a closed, displaced distal fibular fracture in our center. Once the patients met our inclusion criteria (1. unilateral fractures; 2. patients treated with any of the three implants studied in our study; 3. patients with the ability to ambulate without assistance prior injury; 4. Patients who didn’t have osteoarthritis before surgery; 5. patients who had a full one year follow-up data), they were matched by age, BMI and the classifications of the fractures. Based on the different implants used different implants, patients were assigned into three groups: (1) Group A: treated with one-third tubular (Synthes GmbH, Switzerland); (2) Group B: treated with LCP metaphyseal plate (Synthes GmbH, Switzerland); (3) Group C: treated with LCP distal fibula plate (Synthes GmbH, Switzerland) (Figure 2).

Finally 147 patients (49 patients in each group) were included in our study. Trimalleolar fractures were diagnosed in 39 patients, bimalleolar fractures were diagnosed in 88 patients and isolated lateral malleolus fracture was diagnosed in 20 patients. Based on the Weber (AO) classification [10], they were divided into three subgroups: Weber A group: a total of 24 patients (8 patients in each group); Weber B group: a total of 93 patients (31 patients in each group); Weber C group: a total of 30 patients (10 patients in each group).

### Surgical procedure

All the surgeries were performed by the same surgical team (L.L, Z.H) in the same laminar air flow operating room. The team had performed more than 100 surgeries using these implants. Patients were given 1.5 g cefuroxime within 30 minutes prior to skin incision, and general anesthesia was administered in all cases. In Group A, the conventional lateral approach was used while in Group B and C the MIPO technique was used as described by Hess et al. [11]. Tourniquet was placed in all the patients at 100 mmHg above systolic blood pressure. The tourniquet was inflated before incision and deflated after the placement of the implants and then the hemostasis was made before closing the wound.

### Postoperative care

After the surgery, the patients were transferred first to the anesthesia recovery unit for a 2-h period and then to the in-patients unit. Once they were sent to the in-patients unit, cold pack was used on the surgical sites for 12 hours. The drain was removed after 24 hours before being removed. Celecoxib was administered orally with a regular dose of 200 mg bid for pain control regularly after the operation if there was no contraindication. Then it was administered as requested.

In Group A, the patients were mobilized without weight bearing for 6 weeks after the surgery before partial weight bearing was allowed. At the 2 months follow-up weight bearing was initiated after the radiographs being obtained. While in Group B and C, ambulation was started with toe-touch weight bearing of 10 to 15 kg once the soft tissue permitted. Full loading was not allowed until 2 months after the surgery. Patients were followed up in the clinic at 1 month, 2 months, 3 months, 6 months, 1 year after the surgery. Clinical assessment of the soft tissue and ankle function was assessed by a surgeon, while postoperative re-constructive protocols for the patients were done by a physical therapist.

### Outcomes assessment

Clinical outcomes were assessed by both the ankle range of motion (ROM) from the full extension to full flexion and the functional questionnaires including abbreviated Olerud & Molandar Score (OMS) and American Orthopaedic Foot & Ankle Society (AOFAS) clinical rating system [12, 13]. The ROM of each ankle was measured twice in the supine position with a standard (60-cm) goniometer at the time of discharge and every follow-up time points. Details of complications and all the revision surgeries were recorded during the inpatient period and every follow-up time point. Bone healing was defined as follows: (1) pain-free at the location of fracture; (2) three of the four cortices were bridged by visible callus on both the anterioposterior (AP) and lateral view.

Radiographs of the anterioposterior, lateral and mortise view of the involved ankle were obtained preoperatively, postoperatively and at every follow-up time points. Preoperative MRI was taken in order to assess the soft tissue injury, especially the ligament injury. Tscheme classification [14] was used to assess the soft tissue injury. Routine radiographic parameters, including talocrucral angle (TCA) and the medial clear space were measured. The method described by McLennan and Ungersma [15] was used to assess the adequacy of reduction. These measurements were performed by two independent observers. If the no consensus were reached, a senior radiologist was invited to determine the case.

### Statistical analysis

Data management and statistical analysis were performed by SPSS version 18.0 (SPSS Inc, Chicago, IL USA). Shapiro-Wilk test was used to analyze data normality. If the Levene’s test for comparison of variances didn’t reject hypothesis on equality of variance between groups, mean values were compared using ANOVA with Bonferroni correction. Kruskall-Wallis test was applied to non-normally distributed data. The Chi-square test was used to compare the categorical data among the three groups. Analysis was performed with significance level α = .05 (two sided).

## Results

Preoperative patient demographics showed no statistically significant differences among the three groups in terms of age, BMI, sex ratio, smoker ratio or diabetic ratio (Table 1). No significantly statistical difference exists in operation delay, operation time, tourniquet time or plate length among the three groups in different subgroups (Table 2). Totally 10 patients with deltoid ligament injuries were found (3 in Type A and 5 in Type B). Two patients in Type A (1 in Group A, 1 in Group B) and three patients in Type B (1 in each group) needed deltoid ligament repair because of medial instability.

**Table 1:** **Demographic data of the study patients**

Subgroup	Group A	Group B	Group C	P-value
Davis-Weber Type A				
Patients (n)	8	8	8	-
Age (years)	46.9 ± 12.6	47.5 ± 12.0	47.8 ± 13.0	0.990^a^
BMI	22.9 ± 1.1	23.3 ± 1.0	23.2 ± 1.1	0.748^a^
Male (%)	6(75%)	6(75%)	6(75%)	1^b^
Smoker (%)	2(25%)	4(50%)	2(25%)	0.642^b^
Diabetic (%)	0(0%)	2(25%)	0(0%)	0.304^b^
Soft tissue injury				
Tsheme I	3	2	2	1^b^
Tscheme II	5	5	6	
Tscheme III	0	1	0	
Davis-Weber Type B				
Patients (n)	31	31	31	-
Age (years)	47.9 ± 12.9	48.9 ± 14.7	48.5 ± 12.0	0.959^a^
BMI	23.9 ± 1.5	23.8 ± 1.5	23.9 ± 1.5	0.921^a^
Male (%)	17(54.8%)	17(54.8%)	17(54.8%)	1^c^
Smoker (%)	13(41.9%)	12(38.7%)	12(38.7%)	1^c^
Diabetic (%)	2(6.5%)	0(0%)	1(3.2%)	0.77^c^
Soft tissue injury				
Tsheme I	8	11	7	0.810^b^
Tscheme II	21	19	22	
Tscheme III	2	1	2	
Davis-Weber Type C				
Patients (n)	10	10	10	-
Age (years)	46.7 ± 12.0	47.8 ± 11.4	47.4 ± 11.8	0.975^a^
BMI	23.1 ± 2.0	23.2 ± 2.0	23.3 ± 2.1	0.977^a^
Male (%)	6(60%)	6(60%)	6(60%)	1^b^
Smoker (%)	5(50%)	4(40%)	4(40%)	1^b^
Diabetic (%)	1(10%)	0(10%)	0(0%)	1^b^
Soft tissue injury				
Tsheme I	6	7	8	0.668^b^
Tscheme II	4	2	2	
Tscheme III	0	1	0	

Data are presented as mean ± standard deviation (SD) or number with percentage brackets (categorical data).

^a^Data were analyzed using the one-way ANOVA.

^b^Data were analyzed using the Fisher’s exact test.

^c^Data were analyzed using the Chi-square test.

**Table 2:** **Surgical details on the three groups**

Subgroup	Group A	Group B	Group C	P-Value
Davis-Weber Type A				
Operation delay (hrs)	10.0 (5.5-130.0)	9.5 (6.0-106.0)	9.0 (4.75-172.5)	0.983^a^
Operation time (min)	44.4 ± 7.6	47.4 ± 11.0	47.3 ± 11.7	0.804^b^
Tourniquet time (min)	37.1 ± 7.9	41.4 ± 10.9	40.4 ± 11.8	0.696^b^
Plate length (holes)	6	6	6	/
Davis-Weber Type B				
Operation delay (hrs)	8.5 (6.0-148.5)	8.0 (5.5-105.0)	8.0 (6.0-107.0)	0.761^a^
Operation time (min)	44.5 ± 8.1	47.9 ± 8.4	48.5 ± 8.0	0.126^b^
Tourniquet time (min)	36.7 ± 7.8	40.4 ± 7.8	40.2 ± 7.1	0.102^b^
Plate length (holes)	6(6–7)	7(6–7)	7(6–7)	0.068^a^
Davis-Weber Type C				
Operation delay (hrs)	9.5 (4.75-104.0)	8.0 (5.0-80.5)	8.5 (5.0-144.0)	0.987^a^
Operation time (min)	45.9 ± 4.4	47.1 ± 3.9	48.9 ± 5.7	0.375^b^
Tourniquet time (min)	36.4 ± 4.5	39.4 ± 5.1	40.6 ± 6.4	0.222^b^
Plate length (holes)	7(7–8)	7(7–8)	7(7–8)	0.879^a^

Data are presented as mean ± standard deviation (SD) or median with the P_25_ and P_75_ between brackets (numeric data).

^a^Data were analyzed using the Kruskal Waliis Test.

^b^Data were analyzed using the one-way ANOVA.

### Functional outcomes

**Figure 3:**
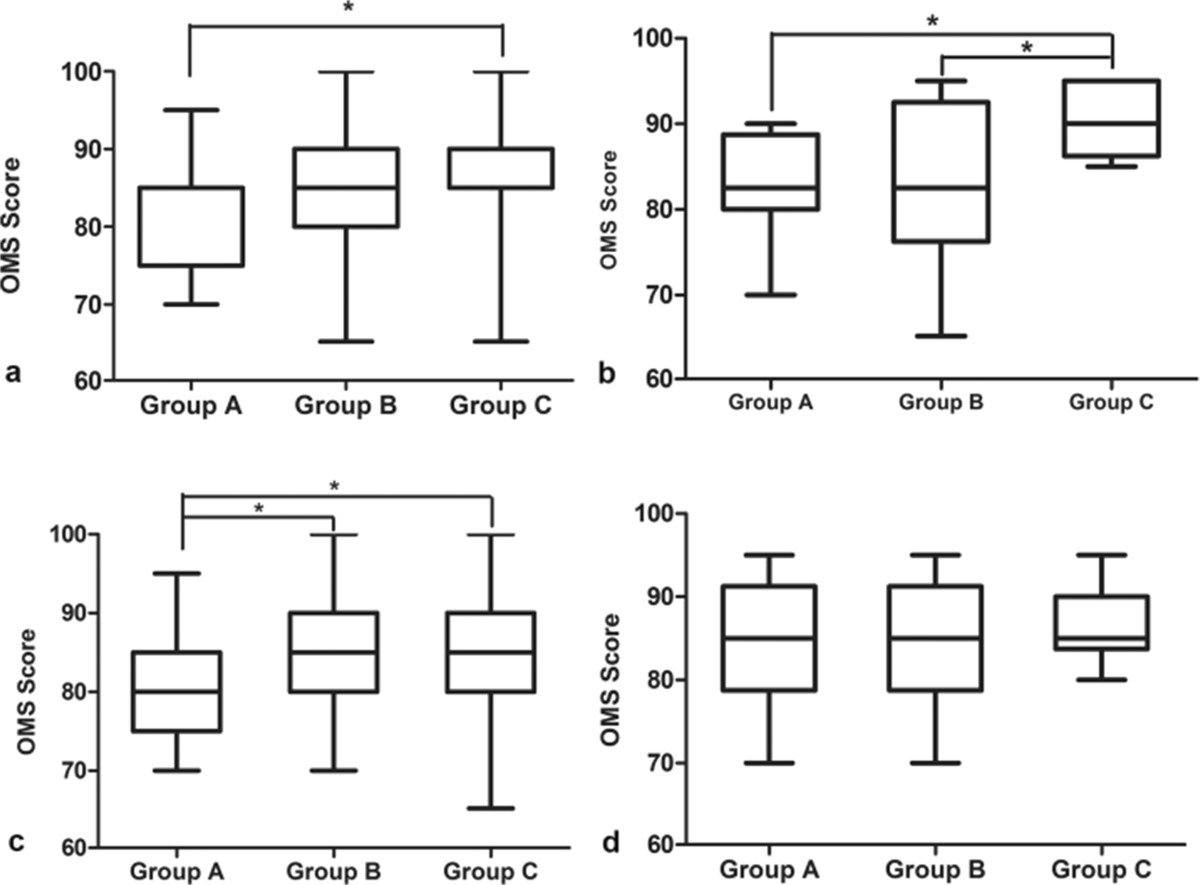
**OMS score at the final follow-up. (a)** All patients; **(b)** Patients with Weber A fracture; **(c)** Patients with Weber B fracture; **(d)** Patients with Weber C fracture. * stands for p < 0.05.

**Figure 4:**
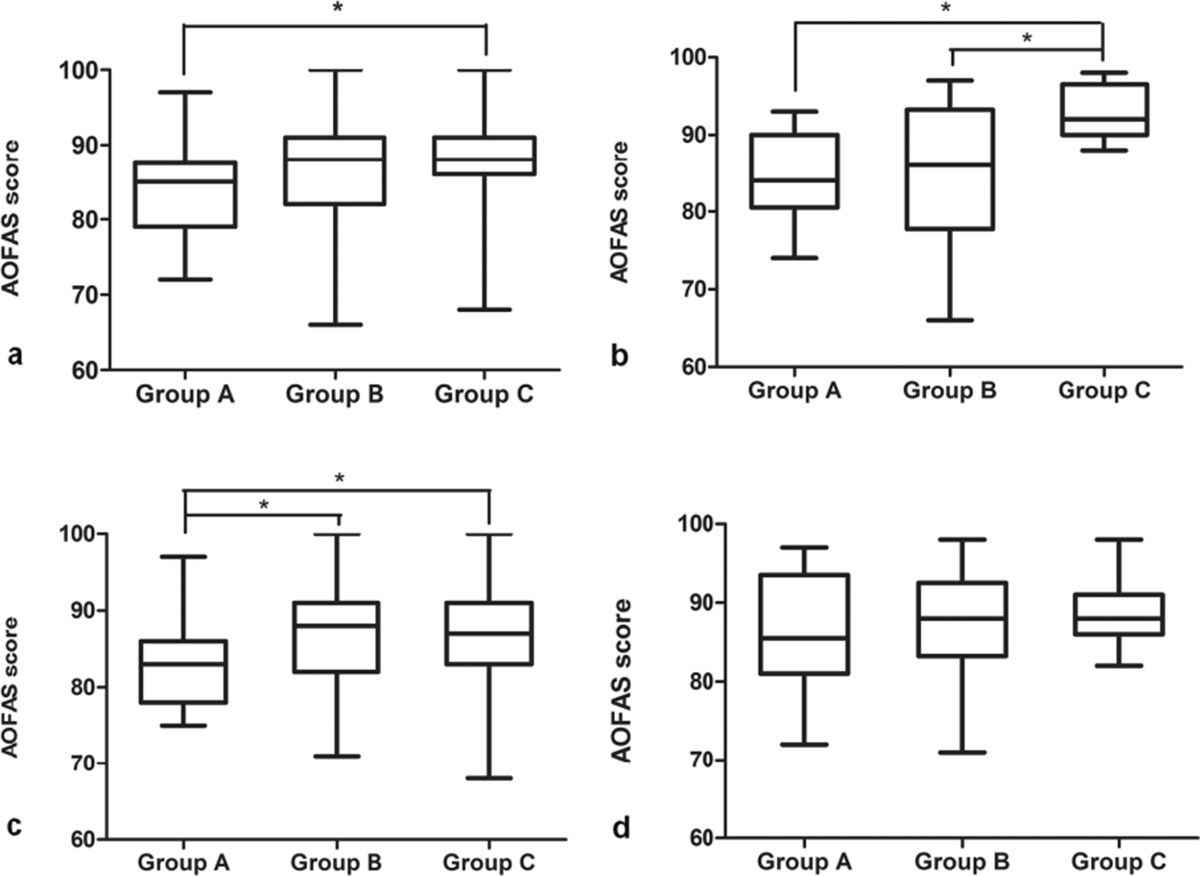
**AOFAS score at the final follow-up. (a)** All patients; **(b)** Patients with Weber A fracture; **(c)** Patients with Weber B fracture; **(d)** Patients with Weber C fracture. * stands for p < 0.05.

At the final 12-month follow-up, multiple comparison analysis of the OMS score showed that: (1) totally, patients in Group C have a significantly better OMS score than those in Group A (86.3 ± 6.2 vs 82.1 ± 6.9, p = 0.004), while no differences were detected between Group B and Group C (Figure 3a). (2) in the patients with Weber A fracture: significant differences were detected between group A and C (82.5 ± 6.5 versus 90.6 ± 4.2, p = 0.02) and also between Group B and C (82.5 ± 8.0 vs 90.6 ± 4.2, p = 0.02), while no statistical differences were found between Group A and B (p = 1.0) (Figure 3b). (3) in the patients with Weber B fracture: significant differences were detected between Group A and B (81.3 ± 6.5 vs 85.2 ± 7.8, p = 0.033) and also between Group A and C (81.3 ± 6.5 vs 85.3 ± 6.7, p = 0.027), while no statistical differences were found between Group B and C (p = 0.928) (Figure 3c). (4) in the patients with Weber C fracture: no statistical differences were detected among the three groups (84.5 ± 8.3 vs 85.0 ± 8.2 vs 86.0 ± 4.6, p = 0.895) (Figure 3d).Multiple comparison of the final follow-up AOFAS score showed: (1) totally, also only significant difference was detected between Group A and Group C (84.0 ± 6.2 vs 88.4 ± 6.9, p = 0.002) (Figure 4a). (2)in the patients with Weber A fracture: statistical differences were detected between Group A and C(84.4 ± 6.1 vs 92.6 ± 3.4, p = 0.029) and also between Group B and C (84.6 ± 10.0 vs 92.6 ± 3.4, p = 0.034), while no statistical differences were found between Group A and B (p = 0.944) (Figure 4b). (3) in the patients with Weber B fracture: significant differences were detected between group A and B(83.2 ± 5.7 vs 86.9 ± 7.5, p = 0.03) and also between Group A and C (83.2 ± 5.7 vs 87.3 ± 6.4, p = 0.017), while no statistical differences were found between Group B and C (p = 0.817) (Figure 4c). (3) in patients with Weber C fracture, also no significant differences were detected among the three groups (86.2 ± 7.9 versus 87.4 ± 8.1 versus 88.3 ± 4.5, p = 0.801) (Figure 4d).

Totally, no significant differences were detected in terms of ROM among the three groups (54.5 ± 9.8°vs 55.6 ± 8.7°vs 55.7 ± 8.6°, p = 0.760). Also significant differences were missing when we did the subgroup analysis (Table 3).

**Table 3:** **ROM and Reduction accuracy**

Subgroup	Group A	Group B	Group C	p^†^	p_1_^†^	p_2_^†^	p_3_^†^
Davis-Weber Type A							
ROM	53.1 ± 9.6	55.0 ± 8.0	56.8 ± 8.0	0.687^a^	0.666^a^	0.392^a^	0.666^a^
Reduction accuracy							
Good	7(87.5%)	7(87.5%)	8(100%)	1^b^	1^b^	1^b^	1^b^
Fair	1(12.5%)	0(0%)	0(0%)	1^b^	1^b^	1^b^	/
Poor	0 (0%)	1(12.5%)	0(0%)	1^b^	1^b^	/	1^b^
Davis-Weber Type B							
ROM	54.4 ± 10.5	55.8 ± 9.1	55.3 ± 9.1	0.832^a^	0.553^a^	0.692^a^	0.843^a^
Reduction accuracy							
Good	28(90.3%)	28(90.3%)	30(96.8%)	0.692^b^	1^b^	0.612^b^	0.612^b^
Fair	3(9.7%)	3(9.7%)	1(3.2%)	0.692^b^	1^b^	0.612^b^	0.612^b^
Poor	0(0%)	0(0%)	0(0%)	/	/	/	/
Davis-Weber Type C							
ROM	56.0 ± 8.8	55.5 ± 8.6	56.0 ± 8.1	0.989^a^	0.896^a^	1^a^	0.869^a^
Reduction accuracy							
Good	9(90%)	8(80%)	9(90%)	1^b^	1^b^	1^b^	1^b^
Fair	1(10%)	2(20%)	1(10%)	1^b^	1^b^	1^b^	1^b^
Poor	0(0%)	0 (0%)	0(0%)	/	/	/	/

Data are presented as mean ± standard deviation (SD) or number with percentage brackets (categorical data).

^a^Data were analyzed using the one-way ANOVA.

^b^Data were analyzed using the Fisher’s exact test.

^†^p stands for p value of Group A VS B VS C, p_1_ stands for p value of Group A VS B, p_2_ stands for p value of Group A VS C, p_3_ stands for p value of Group B VS C.

### Radiographic outcomes

Only one patient associated with a Type A fracture in Group B was confirmed with a poor reduction accuracy by postoperative 3D-CT scan. No statistical significant was achieved among the three groups of any subgroups in terms of reduction accuracy (Table 3). The healing time was significantly less in patients of Group C than those in Group A (20.0 ± 3.8w vs 23.1 ± 3.6w, p < 0.0001) and Group B (20.0 ± 3.8w vs 23.0 ± 3.4w, p < 0.0001). While no significant difference was found between Group A and Group B (p = 0.867).

### Complications

One patient with the Weber A fracture treated with one-third tubular plate was diagnosed superficial infection clinically during the inpatient period. He was treated with regular dressing change and intravenous antibiotics. The infection was controlled avoiding hardware removal. At the final follow-up, the patient had a ROM of 50°. One patient of Group B with Weber A fracture was found nonunion. No deep infection or loss of reduction was found in the 147 patients until the final follow-up. None developed superficial peroneal nerve injury.

## Discussion

The closed and displaced distal fibular fractures usually occur in a relative young and active population as a result of minor trauma [16]. As these people have a high request of the activity, it is quite essential to regain the length of the fibula and maintain the stability of the lateral malleolus. Thus, surgical treatment is used as a standard practice.

The goal of our study was to compare our clinical results with one-third tubular, LCP metaphyseal plate and LCP distal fibula plate. The most important finding of the present study was that the functional scores of patients treated with LCP distal fibula plate were significant higher than those treated with one-third tubular plate. The healing time of patients in Group C was significant less than those in Group A and Group B. The subgroup analysis showed that: (1) In subgroup of Weber A, the functional scores of the Group C were higher than those in Group A and B. (2) In subgroup of Weber B, the functional scores of the Group B and C were much higher than those in Group A. (3) In subgroup of Weber C, no statistical differences was found among the three groups. In terms of the ROM and reduction accuracy no differences existed among the three groups. Superficial infection was diagnosed in one patient with Weber A fracture treated with one-third tubular plate. One nonunion was found in one patient of Group B with Weber A fracture.

Using of locking plates and minimally invasive technique is a suggested alternative to the traditional lateral plating techniques. Compared with the traditional one-third tubular plate, the LCP plates can provide more stable fixations and much earlier range of motion exercise [17] which has been proved by several vitro biomechanical studies [18, 19]. In comparison with the LCP metaphyseal plate, distal part of the LCP distal fibula plate has an anatomical expansion which can provide more holes of multiple screw choices. Kim et al. [20] reported in their biomechanical experiment with locking plates in distal fibula that, 2 distal unicortical locking screws are mechanically equivalent to a standard plate with 3 distal screws. By this kind of distal expansion design, using the LCP distal fibula plate can provide a better implant bone match and a better distal stability for distal fragments of the Weber A fracture, which is quite vital to delaying or avoiding the occurrence of the traumatic osteoarthritis. This might account for why the LCP distal fibula plate is superior to the other two implants in the patients with Weber A fracture in terms of both OMS and AOFAS scores. When it comes to the Weber B fracture, two kinds LCP plates can provide a more stable bridge, which allows the patients to have an earlier functional exercise in order to regain a better walk ability.

As to the ROM, the mean value of the three groups in different subgroups did not show any significant difference though the patients with Weber A or B fracture treated with one-third tubular plate have a relatively lower ROM than the other patients. We believe there might be some reasons for this phenomenon. First, both OMS and AOFAS are general scales which evaluate many aspects of the foot, including pain, function, gait and alignment. So the difference in function score might not present the difference in the ROM between the groups. Second, we performed the standard postoperative rehabilitation program in all the patients. Early function rehabilitation was emphasized in our daily medical practice, the physical therapist at clinic would give more focus on those patients who had a relatively poor ROM. Third, in this study, we compared the functional scores at the 12-month follow-up. As we know, the functional rehabilitation usually stabilizes at the 6-month after the surgery. So, the difference at the early stage might not be detected.

The MIPO technique was developed so as to prevent periosteal devascularizatiuon and major soft tissue dissection. This technique not only allows inserting a plate as an internal fixation through a small incision, but also protects both the skin and the fracture fragments. Cadaver studies have indicated that this technique is superior to the traditional one in terms of preserving the para-femur vessels [21, 22]. Clinical studies evaluating this technique for fractures of the long bones such as the femur, tibia, and humerus illustrated an accurate bone healing rate with few complications, at least comparable to the traditional ORIF [23, 24]. However, few studies have ever evaluated the feasibility and the results of the MIPO technique for the distal fibula fracture [11]. To authors’ knowledge, this study is the first one to compare the MIPO technique with the traditional one for the distal fibula fracture. We have observed no soft tissue related complications in the patients treated with the MIPO technique, while one patient treated with the traditional one was diagnosed as superficial infection. Moreover, no nerve injury was observed in this present study, though we didn’t explore the superficial peroneal nerve during the surgery. There might be four reasons as follows: firstly, we carefully checked the length of the plate preoperatively on the 100% radiographs and intraoperatively prior to the insertion with both the proximal and distal incision marked on the skin; secondly, after the dissection, the periosteal elevator was used to prepare the extra-peropsteal tunnel for the incision of the plate from both proximal and distal incision; thirdly, during the final positioning of the plate and screw insertion, careful attention was paid when we retracted the soft tissue as not to cause any injury to the nerve; fourthly, the length of the plate used in our study was no longer than 8-holes, and according to Neubauer et al. [25]’s study the nerve injury occurs a lot within the proximal 4 holes of the longer 10-holes plate.

Overall, anatomical reduction was achieved in 134 patients (91.2%). The rate of anatomical reduction is comparable among the traditional plate LCP groups. After analyzing the only nonunion we observed, we thought it might lie in the following reasons. Firstly, this patient had a comminuted Weber A fracture with an extreme soft tissue swelling. At the time of fixation, the LCP metaphyseal plate could not provide an enough cover of the distal fibula, leaving no ample reduction of the fragments. Secondly, after the discharge the diabetes was not well controlled with a postprandial glucose of more than 20 mmol/L. After the X-ray and CT scan had confirmed the nonunion, we used the LCP metaphyseal plate to refixed the fracture with local autogenous bone graft. The rate of complication was quite lower than that in Schepers et al.’s [26] study. The reasons might be as follows: first, we excluded the open fractures, according to Miller et al.’s [27] study, the wound outcome after ankle surgery has nothing to do with the time to the surgery but with open fracture. Secondly, the patients included in our study followed the postoperative instructions very well, so that any minor wound conditions were treated in time.

There are limitations to this study. Although these data were collected in our prospective database, it was a retrospective analysis of available information. We included the patients with enough follow-up information and excluded the others, which might cause potential selection. We didn’t calculate the needed sample size before the study. The number of patients with Weber A and C fracture was relatively small. A larger sample size might be needed to detect significance in assessment outcomes among the groups.

However, strength of our study is that patients were matched by age, BMI and divided into subgroups according to different classifications of fractures which powers it to suggest: (1) Using LCP plates with MIPO technique for the distal fracture can achieve good outcomes; (2) LCP metaphyseal plate shows superior to the other two plates for the patients with Weber A fracture in functional outcomes; (3) As for the Weber B fracture, the LCP plates shows advantages over the traditional plate.

## Conclusion

This study demonstrated using LCP metaphyseal plate in patients associated with lateral malleolar fracture could achieve significantly better OMS & AOFAS scores and less healing time than using one-third tubular plate. Specifically, For Weber A fracture, LCP distal fibula plate is much better than one-third tubular plate and LCP metaphyseal plate. While for Weber B fracture, LCP distal fibula plate and LCP metaphyseal plate are better than one-third tubular plate. As to the complications, using MIPO technique in patients with distal fibular fractures is at least comparable to the traditional one. High-quality randomized controlled trials are needed to certify our findings in the future.

## Authors’ original submitted files for images

Below are the links to the authors’ original submitted files for images. Authors’ original file for figure 1 Authors’ original file for figure 2 Authors’ original file for figure 3 Authors’ original file for figure 4

## Abbreviations

MIPO: Minimally invasive plate osteosynthesis
LCP: Locking compression plate
OMS: Olerud & Molandar Score
AOFAS: American Orthopaedic Foot & Ankle Society score.
