# Supplementary figures and images for: Comparison of three plate system for lateral malleolar fixation
Source: BMC Musculoskelet Disord. 2014 Oct 30;15:360. doi: 10.1186/1471-2474-15-360 (PMC4223732; doi:10.1186/1471-2474-15-360)

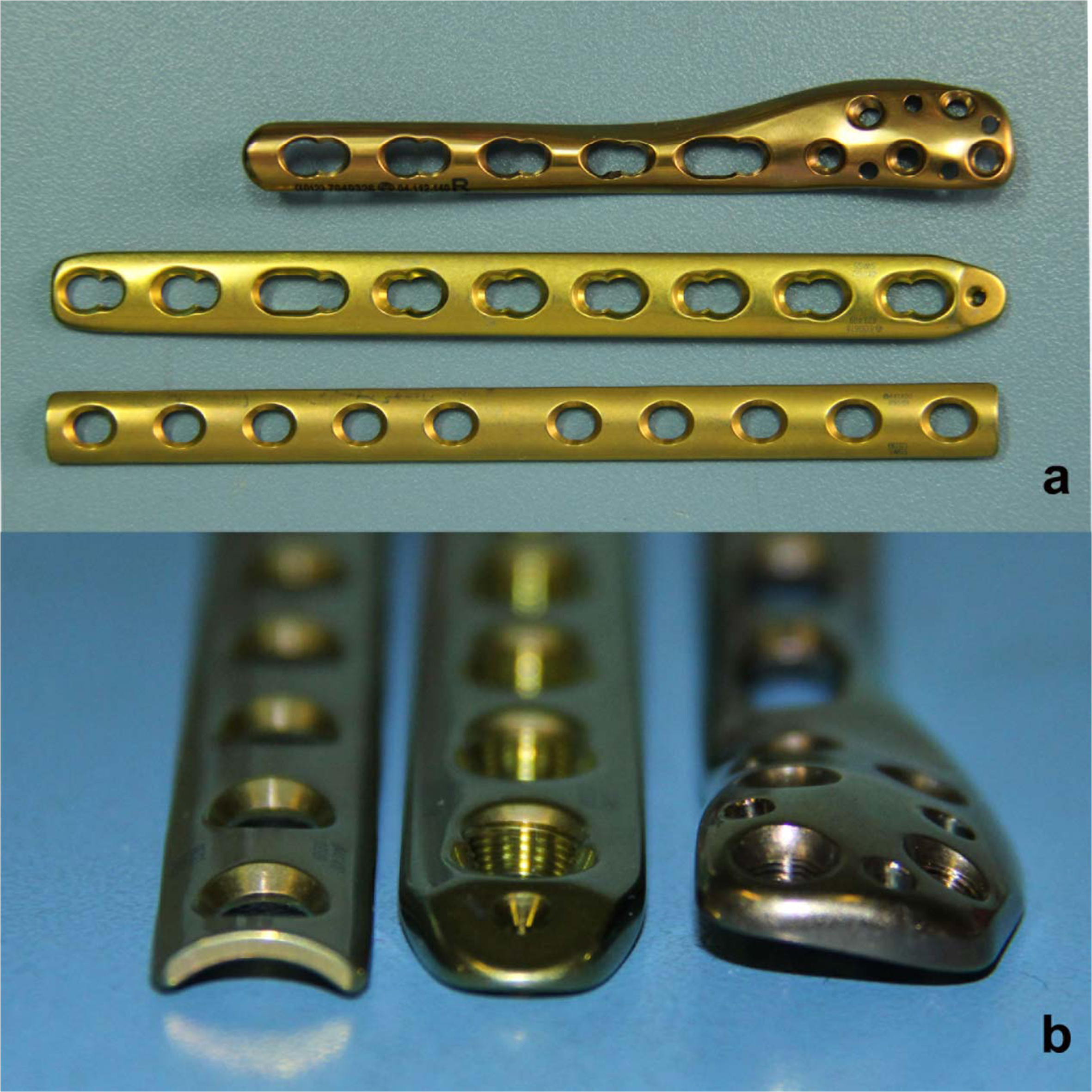

Supplement: Supplementary file 1 — Authors’ original file for figure 1 [file 12891_2013_2295_MOESM1_ESM.tif]

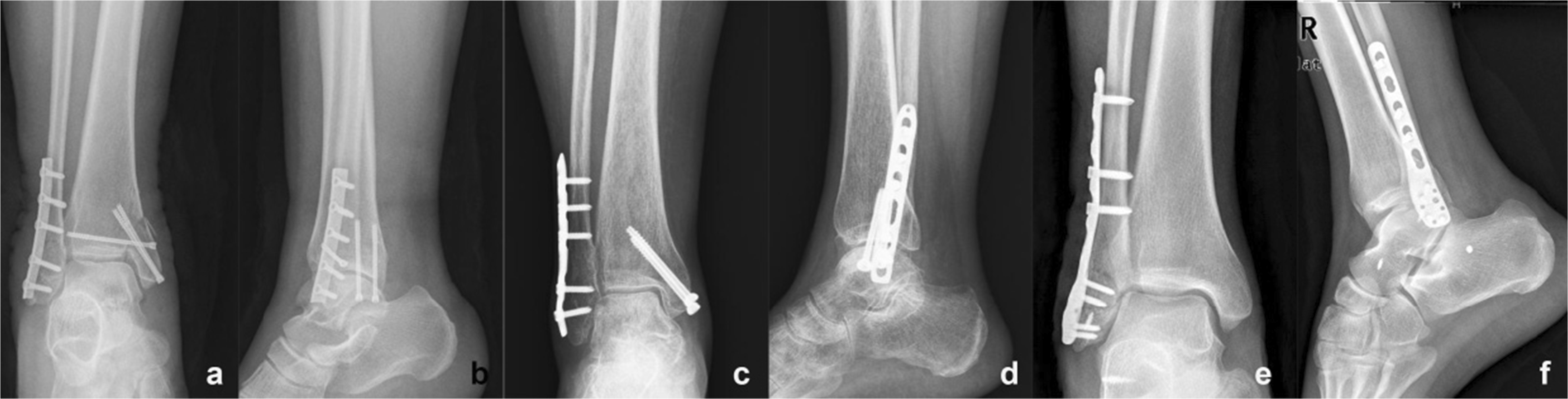

Supplement: Supplementary file 2 — Authors’ original file for figure 2 [file 12891_2013_2295_MOESM2_ESM.tif]

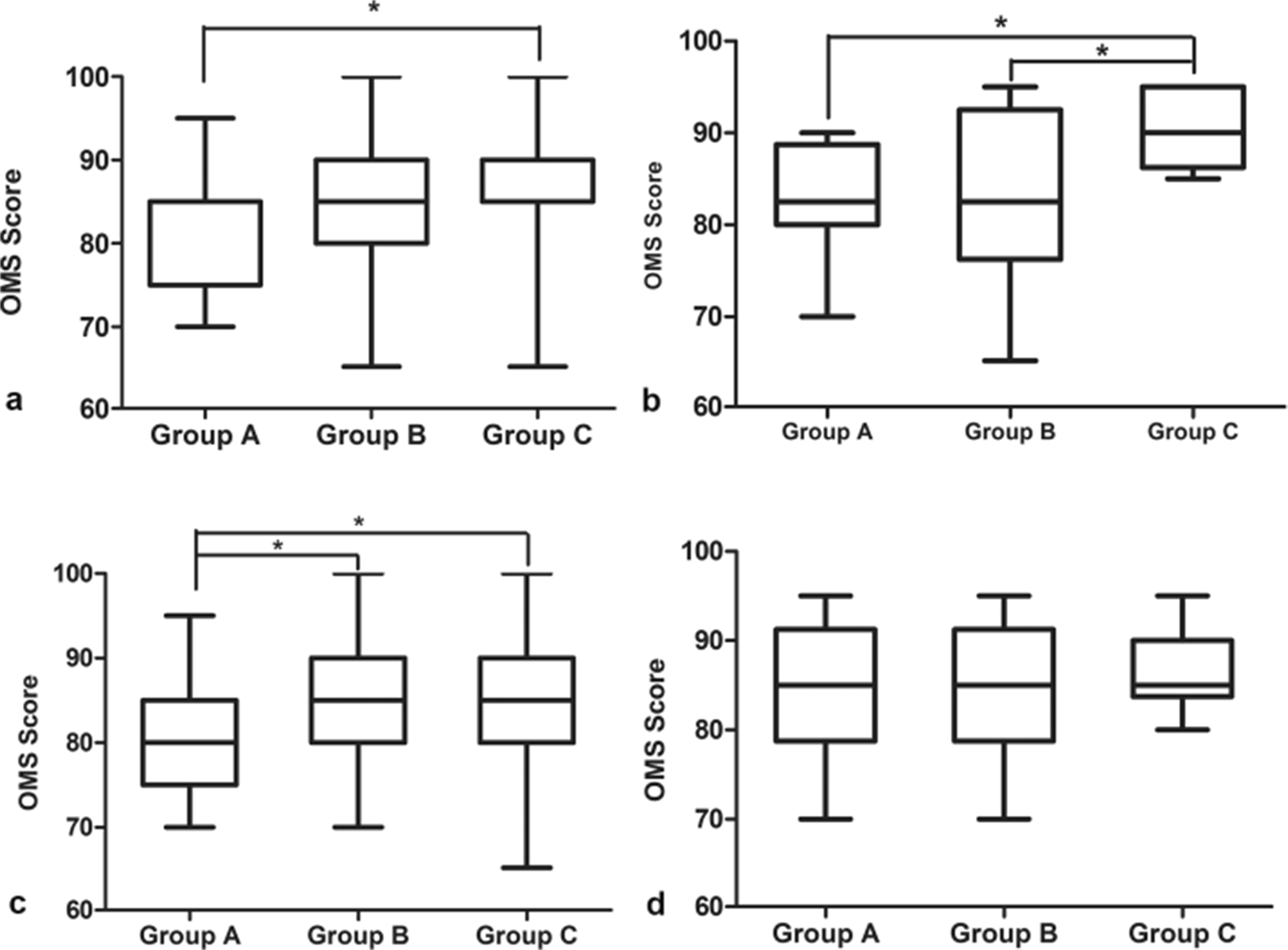

Supplement: Supplementary file 3 — Authors’ original file for figure 3 [file 12891_2013_2295_MOESM3_ESM.tif]

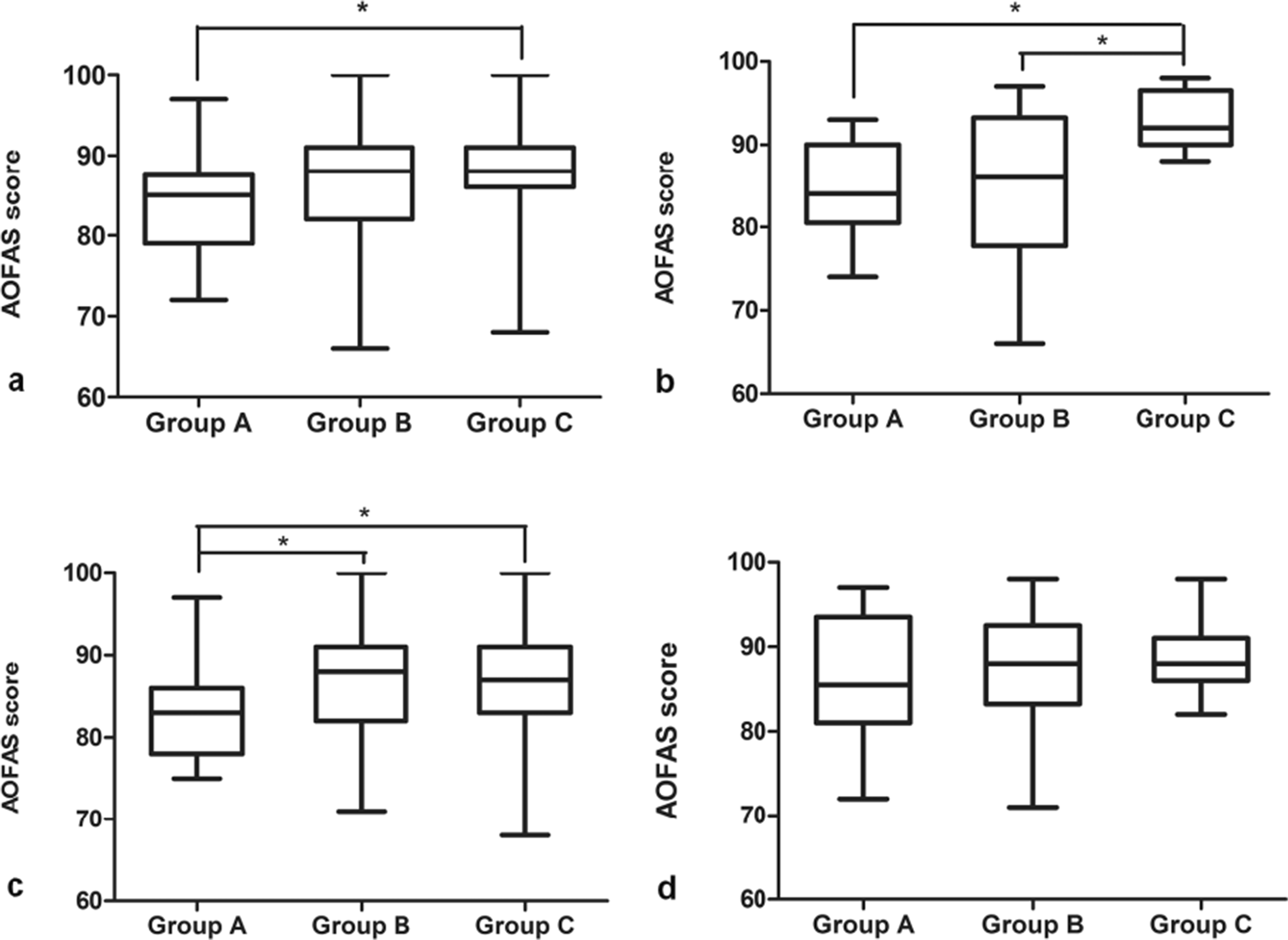

Supplement: Supplementary file 4 — Authors’ original file for figure 4 [file 12891_2013_2295_MOESM4_ESM.tif]
